# Bariatric Surgery Improves the Cavernosal Neuronal, Vasorelaxation, and Contraction Mechanisms for Erectile Dysfunction As Result of Amelioration of Glucose Homeostasis in a Diabetic Rat Model

**DOI:** 10.1371/journal.pone.0104042

**Published:** 2014-08-19

**Authors:** Yong Sun Choi, Sang Kuon Lee, Woong Jin Bae, Su Jin Kim, Hyuk Jin Cho, Sung-Hoo Hong, Ji Youl Lee, Tae-Kon Hwang, Sae Woong Kim

**Affiliations:** 1 Department of Urology, College of Medicine, The Catholic University of Korea, Seoul, Korea; 2 Department of Surgery, College of Medicine, The Catholic University of Korea, Seoul, Korea; The Chinese University of Hong Kong, Hong Kong

## Abstract

**Background:**

Bariatric surgery is an effective treatment option for both obesity and obesity-related type 2 diabetes mellitus (T2DM). However, little is known regarding the effects of bariatric surgery on erectile dysfunction among patients with T2DM. Therefore, we investigated whether bariatric surgery would lead to structural and biochemical changes in the corpus cavernosum.

**Material and Method:**

Twenty-five male Otsuka Long-Evans Tokushima Fatty rats were assigned to either a control group (sham operation, n = 10) or a bariatric surgery group (gastric bypass surgery, n = 15). Four weeks after the operation, each group of rats was evaluated with an oral glucose tolerance test (OGTT). The penile intracavernous pressure was measured for erectile functional analysis. Histologic evaluation of the tissue was performed with Masson's trichrome staining. Endothelial nitric oxide synthase (eNOS), neuronal nitric oxide synthase (nNOS), Rho kinase, and 8-hydroxy-2-deoxyguanosine (8-OHdG) levels in the corpus cavernosum were assayed by using western blot and ELISA.

**Results:**

The mean body weight of the bariatric surgery group was lower than the control group (p = 0.002). The postoperative OGTT result was lower in the bariatric surgery group than in the control group (p = 0.014), and this was lower than the preoperative value (p = 0.037). The intracavernous pressure/mean arterial pressure ratio was higher in the bariatric surgery group compared to the control group (p = 0.021), and a higher cavernosum smooth muscle/collagen ratio was observed in the bariatric surgery group compared to the control group (p = 0.025). Likewise, the expression of eNOS and nNOS was higher in bariatric surgery group than in the control group (p = 0.027 and p = 0.008, respectively). Decreased expression of Rho kinase and levels of 8-OHdG were observed in the bariatric surgery group (p = 0.032).

**Conclusion:**

In this animal model, bariatric surgery appears to ameliorate T2DM-related metabolic dysfunction leading to structural and biochemical changes in the corpus cavernosum, and thus, results in improvement of erectile dysfunction associated with T2DM.

## Introduction

Obesity and type 2 diabetes mellitus (T2DM) are associated with cardiovascular disease, cerebral disease, and metabolic diseases such as hyperlipidemia and erectile dysfunction (ED) [1]–[3]. The socioeconomic burden of these diseases is huge and on the rise [2], [4]. These diseases are the result of macro- and microvascular endothelial dysfunction. Various treatment options are available such as life-style modifications, proper nutritional management, and oral medications. However, bariatric surgery is considered the most effective treatment modality for obesity and T2DM when considering the long-term results of each intervention [5]–[7]. Because T2DM and obesity are closely linked, bariatric surgery for the treatment of obesity can improve glucose tolerance. As such, there are a few reports suggesting that bariatric surgery can improve diabetic complications including sexual dysfunction in obese men [8].

Microvascular injury and corpus cavernosal fibrosis are considered the main pathophysiological contributors to T2DM- or obesity-related erectile dysfunction. Bariatric surgery may correct metabolic abnormalities such as glucose intolerance, and thereby reduce microvascular disease and improve erectile dysfunction. In spite of these advantages of bariatric surgery, little is known about the effects or mechanism of bariatric surgery on erectile dysfunction in patients with diabetes.

Therefore, in this study, we investigated how bariatric surgery could lead to the improvement of diabetic and obesity-related erectile dysfunction by examining the structural and biochemical changes of the corpus cavernosum in the Otsuka Long-Evans Tokushima Fatty (OLETF) rat model.

## Materials and Methods

### Ethics Statement

This study was reviewed and conducted under the Institutional Animal Care and Use Committee at the School of Medicine of The Catholic University of Korea. The Institutional Review Board approval number was CUMS 2010-0125-01.

### Experimental Animals

Male OLETF rats (n = 25) aged 25–29 weeks were obtained for the study. After one week of acclimatization, each rat was randomly assigned to one of the two study groups. Those assigned to the control group underwent a sham operation (n = 10). The rats assigned to the bariatric surgery group underwent gastric bypass surgery (n = 15). Preoperative weight measurement and fasting glucose tolerance tests were performed in all rats.

### Surgical procedures

In the bariatric surgery group, duodenal-jejunal bypass (DJB) surgery was performed according to the details published by F. Rubini et al. [5] The entire duodenum with 10 cm of the proximal jejunum was divided and bypassed from the stomach. During the operation the stomach volume was maintained. Then, the gastrojejunal anastomosis was performed at 10 cm distal to the ligament of Treitz. The bibliopancreatic secretions were drained from the reconstructed biliary limb to the alimentary limb of the small bowel in Roux-en-Y fashion. Because the length of small bowel in rats has a length of approximately 100 cm, this procedure consists of a similar technique and functional outcomes as a stomach-sparing, proximal, Roux-en-Y bypass surgery in humans [5].

The control group underwent a sham operation, in which the gastrointestinal tract was transected and reanastomosed to maintain the physiologic circuit of food through the bowel.

### Oral Glucose Tolerance Test

Four weeks after the operation, an oral glucose tolerance test was performed. To establish a baseline fasting glycemia, peripheral blood was collected from the rat tail after a 12- to 14-hour fasting period. Blood samples were centrifuged, and the plasma glucose was measured with a glucometer. Blood samples collected at 30, 60, 90, and 120 minutes after the glucose gavage and analyzed to measure plasma glucose response.

### Measurement of Erectile Function

Four weeks after the operation, rats were anesthetized with Zoletil (50 mg/kg) and placed on a table in the supine position to measure the mean arterial pressure (MAP) and intracavernosal pressure (ICP). For MAP measurement, PE-50 tubing was inserted in the carotid artery, and the ICP was measured through a 23-gauge butterfly needle in the corpus cavernosum that was connected to a pressure transducer (Grass Model S48K, Grass Instrument Division, Astro-Med Inc., West Warwick, RI). The cavernous nerve stimulation was administered with a bipolar stainless electrical stimulator during 50 seconds at 10 V and 2.4 mA with a pulse width of 0.5 milliseconds. The maximal amplitude of ICP during the cavernous nerve electrostimulation was analyzed. The ratio of ICP/MAP was calculated for the determination of erectile function [6].

### Histological Analysis of Corpus Cavernosum

Each rat was euthanized at four weeks post-surgery after repeated weight measurements, OGTT, and erectile function measurement. The middle section of the penile shaft was denuded and fixed in 10% formalin overnight. After washing, the tissue was stored in 70% alcohol at 4°C for paraffin-embedded tissue sectioning, then stained with Masson's trichrome stain. Digital microscopic photographs were obtained, and the color distribution was calculated with image software (Image Pro Plus, ver. 5.0, Media Cybernetics, Rockville, MD 20850, USA). The proportion of muscle/collagen fiber ratio was evaluated three to five times for each specimen, and was expressed as the mean ± standard deviation.

### Western Blot Analysis

For each corpus cavernosum tissue sample, a 20-mg piece was cut into smaller segments. Following this, 250 µL RIPA buffer (25 mM Tris-HCl (pH 7.6), 150 mM NaCl, 1% NP-40, 1% sodium deoxycholate, 0.1% SDS, protease inhibitor cocktail) was added to the tissue. This resulting product was homogenized on ice and incubated at 4°C for 16 hours. The samples were centrifuged at 13,000 rpm, at 4°C, for 15 minutes and the supernatant was transferred to new tubes. It was then quantified with the Bradford protein assay (Bio-Rad, CA, USA) and stored at −70°C.

Using an 8%-15% SDS-polyacrylamide gel, 50 µg of the proteins were electrophoresed at 100 V for 1.5 hours. The protein bands were then transferred to PVDF membranes (Millipore, Billerica, USA) at 70 V for 2 hours, blocked with 5% skimmed milk solution for 1 hour, then incubated with eNOS (1∶500, BD Pharmingen, San Diego, CA), nNOS (1∶4000, BD Pharmingen), Rho kinase, or beta–actin (1∶10000, SantaCruz, CA, USA) at 4°C for 16 hours. Membranes were then washed 3 times with TBST solution and incubated with anti-mouse IgG-HRP (1∶2000, 1∶5000 Invitrogen, La Jolla, CA) and anti-rabbit IgG-HRP (1∶2000, Invitrogen) at room temperature for 1 hour. Membranes were washed with TBST solution for 10 minutes three times, reacted with ECL plus solution (GE Healthcare, UK) for 1 minute, then were exposed on film. The intensity of the bands was compared and the expression of proteins including the differences between the control and experimental groups were analyzed.

### Oxidative Stress Analysis

The oxidative stress for each corpus cavernosum tissue was analyzed by quantifying the levels of 8-OHdG as a measure of oxidatively modified DNA. Using the DNeasy Blood and Tissue kit (Qiagen, Valencia, CA), the total DNA was extracted from the corpus cavernosum according to the manufacturer's protocol.

The 8-OHdG levels were measured with a DNA oxidation kit (Highly Sensitive 8-OHdG Check ELISA; Japan Institute for the Control of Aging, Fukuroi, Japan) according to the manufacturer's protocol. The extracted DNA was incubated for 1 hour with anti 8-OHdG monoclonal antibody in a microtiter plate precoated with 8-OHdG, and compared against 8-OHdG standards of known concentrations (0.5–40 ng/mL). After the ELISA had fully developed, the absorbance at 450 nm was measured on a spectrophotometer and 8-OHdg concentrations were quantified. Tissue sample concentrations were calculated by using the standard curve and were corrected for DNA concentration.

### Statistical Analysis

Data are expressed as the mean ± standard deviation. Statistical analysis was performed by using the Mann Whitney U test and SPSS 19.0 (IBM Software NY, US), and p values<0.05 were considered to be statistically significant.

## Results

### Body weight

**Figure 1:**
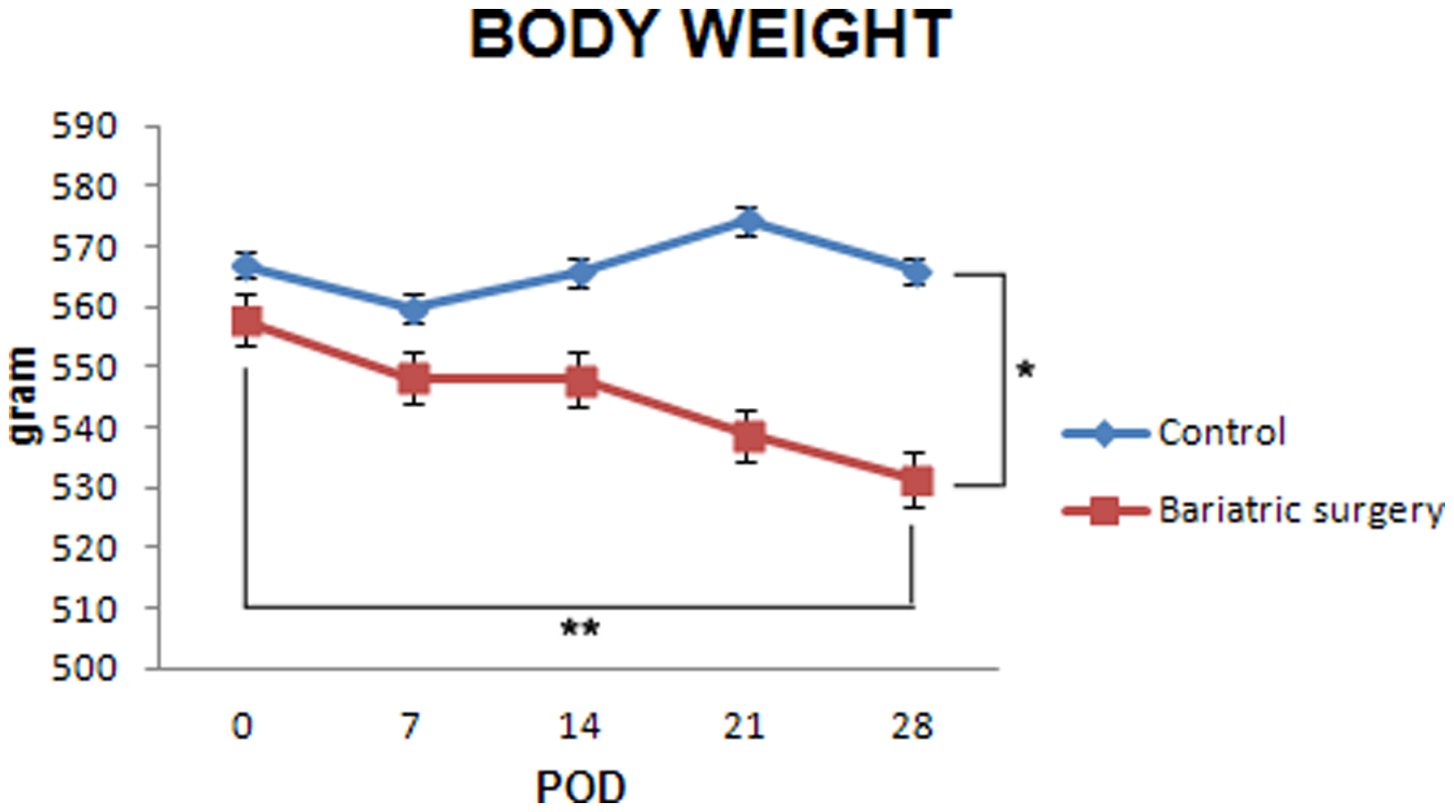
Body weight of type 2 diabetes mellitus (T2DM) rats after bariatric surgery (*: p = 0.002, **: p = 0.007).

The mean preoperative body weight of the control and bariatric surgery groups was 567.1±52.1 g and 558.0±61.1 g, respectively. Four weeks after the operation, the mean body weight of the control and bariatric surgery groups was 566.1±63.1 g and 531.5±75.2 g, respectively. The body weight at 4 postoperative weeks in the bariatric surgery group was lower than preoperative values (p = 0.007) and the final mean body weight of the bariatric surgery group was lower than the control group (p = 0.002) (Fig. 1).

### Oral Glucose Tolerance Test

**Figure 2:**
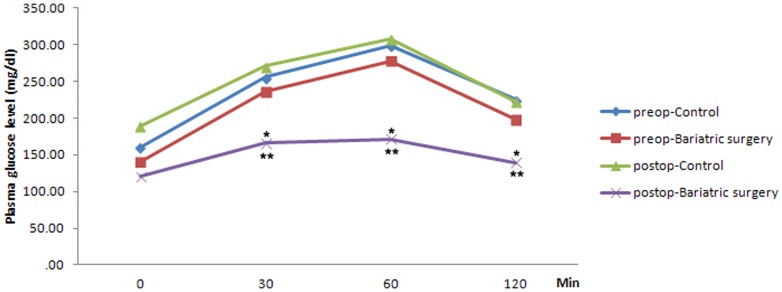
Oral glucose tolerance test before and after bariatric surgery in rats (*: compared with preoperative bariatric surgery, p = 0.037 **: compared with postoperative control group, p = 0.014).

The glucose tolerance test was performed pre- and post-surgery. The mean preoperative OGTT levels in the control and bariatric surgery groups were 160.8±17.7 mg/dL and 140.6±21.2 mg/dL, respectively. Prior to surgery, a glucose loading test was performed and the glucose levels after 120 minutes in the control and bariatric surgery groups were 225.2 mg/dL and 198.2±12.2 mg/dL, respectively. Following surgery, the fasting glucose levels in the control and bariatric surgery groups were 189.1±48.2 mg/dL and 121±20.6 mg/dL, respectively. After the glucose loading test was performed, the glucose levels after 120 minutes in the control and bariatric surgery groups were 222.5±66.4 mg/dL and 139.5±10.9 mg/dL, respectively (Table 1). Glucose intolerance did not change in the control group rats, while the rats in the bariatric surgery group showed improvement in glucose tolerance. The postoperative OGTT level in the control group was not different from the preoperative OGTT level, suggesting that surgical stress and food intake did not affect glucose tolerance. Compared with the preoperative OGTT level, the postoperative OGTT level in the bariatric surgery group showed a marked improvement in glucose tolerance (198.2±12.2 mg/dL vs. 139.5±10.9 mg/dL, p = 0.037). Furthermore, the postoperative OGTT plasma glucose level was lower in the bariatric surgery group compared to the control group (222.5±66.4 mg/dL vs. 139.5±10.9 mg/dL, p = 0.014) (Fig. 2).

**Table 1:** Preoperative and postoperative oral glucose tolerance test (OGTT).

	Minute	Control	Bariatric Surgery	p-value
Preoperative OGTT	0	160.8±17.7	140.6±21.2	0.067
	30	255.9±58.8	236.4±45.7	0.074
	60	298.5±67.3	277.6±62.5	0.059
	120	225.2±53.9	198.2±12.2	0.052
Postoperative OGTT	0	189.1±48.2	121.0±20.6	0.038
	30	270.7±72.1	166.0±22.2	0.021
	60	307.3±83.8	171.4±16.4	0.009
	120	222.5±66.4	139.5±10.9	0.014

OGTT results in the control and bariatric surgery groups before and after the operation. The preoperative OGTT was measured before the operation and the postoperative OGTT was measured 4 weeks after the operation.

### Measurement of Erectile Function

**Figure 3:**
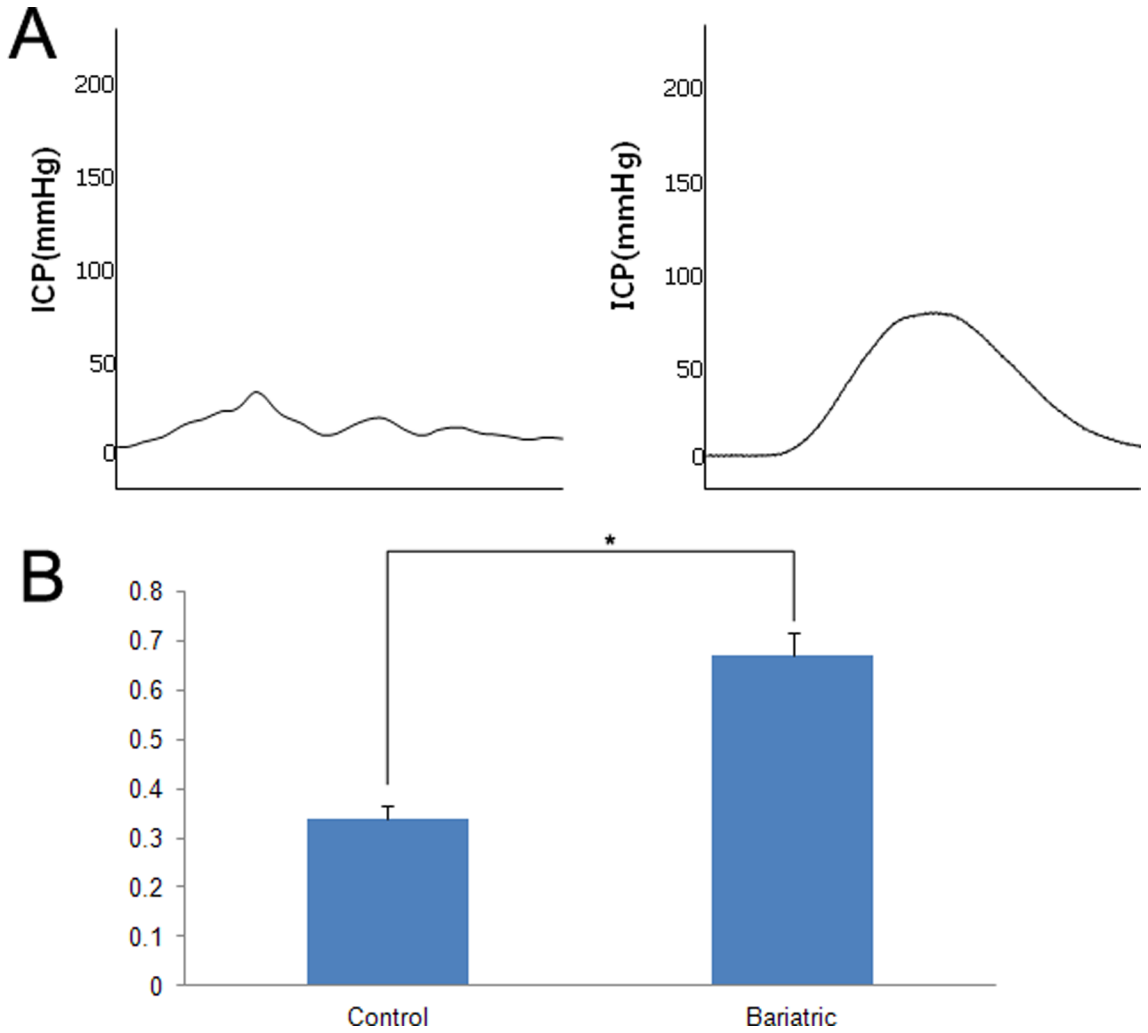
Erectile function result at 4 weeks after surgery. (**A**) Representative recordings of intracavernosal pressure (ICP) for the control and bariatric surgery groups in response to cavernous nerve stimulation at 10 V. (**B**) The ratio of ICP/mean arterial pressure (MAP) was calculated and compared for each group. (*: p = 0.021).

To assess penile erectile function, the ratio of ICP/MAP was measured in response to cavernous nerve electrostimulation. The MAP results from the control and bariatric surgery groups were 142.4±11.5 mmHg and 129.7±13.2 mmHg, respectively. The maximum ICP value during electrostimulation in the control and bariatric surgery groups were 48.7±8.8 mmHg and 87.5±10.2 mmHg, respectively (Fig. 3A). The ICP/MAP ratios in the control and bariatric surgery groups were 0.34±0.05 and 0.67±0.07, respectively. The bariatric surgery group showed an increase in the ICP/MAP ratio (p = 0.021) (Fig. 3B).

### Histological Analysis of Corpus Cavernosum

**Figure 4:**
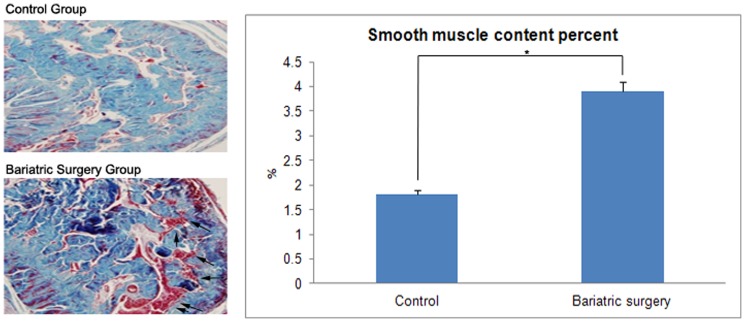
Histological analysis and smooth muscle/collagen fiber ratio between the control and bariatric surgery groups. The muscle fibers stained with red color were marked with an arrow. (Magnification ×100, *: p = 0.025).

In the histological analysis of the corpus cavernosum tissue, a marked reduction of smooth muscle fibers and increased collagen fibers was demonstrated in the control group, whereas the volume of smooth muscle fiber was relatively preserved in the bariatric surgery group and was higher than in the control group. The smooth muscle/collagen fiber volume ratio was 1.81±1.4 in the control group and 3.9±1.5 in the bariatric surgery group (p = 0.025) (Fig. 4).

### Western Blot Analysis

**Figure 5:**
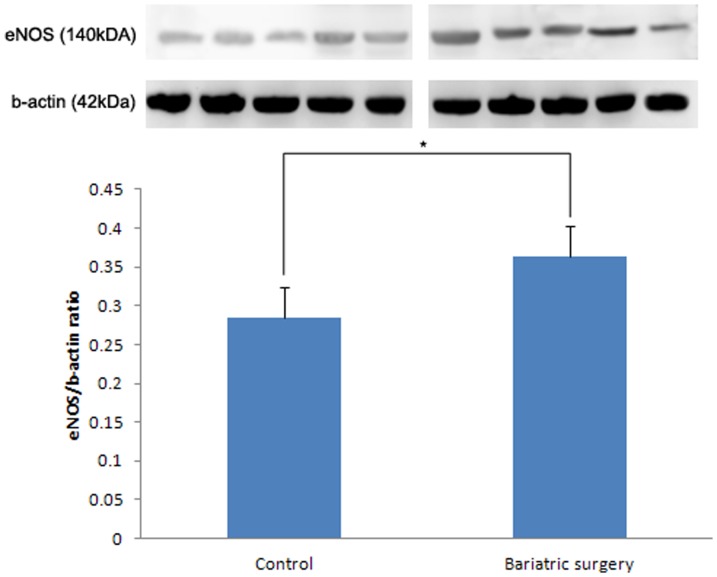
eNOS/b-actin ratio in the control and bariatric surgery groups (*: p = 0.027).

**Figure 6:**
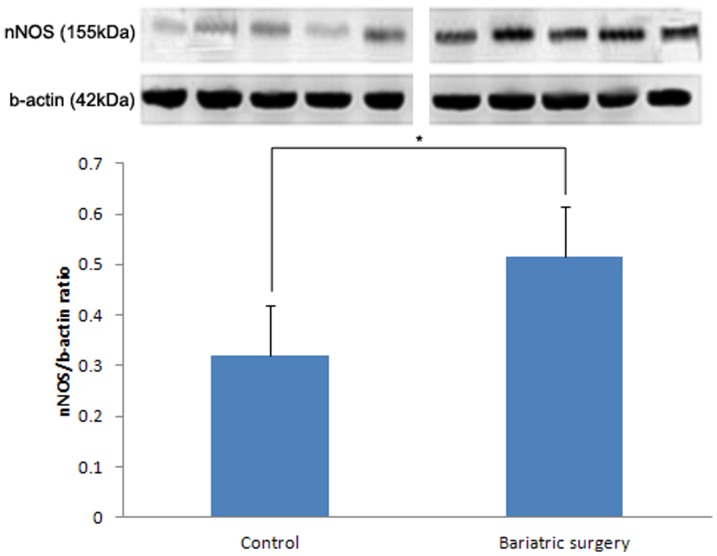
nNos/b-actin ratio in the control and bariatric surgery groups (*: p = 0.008).

**Figure 7:**
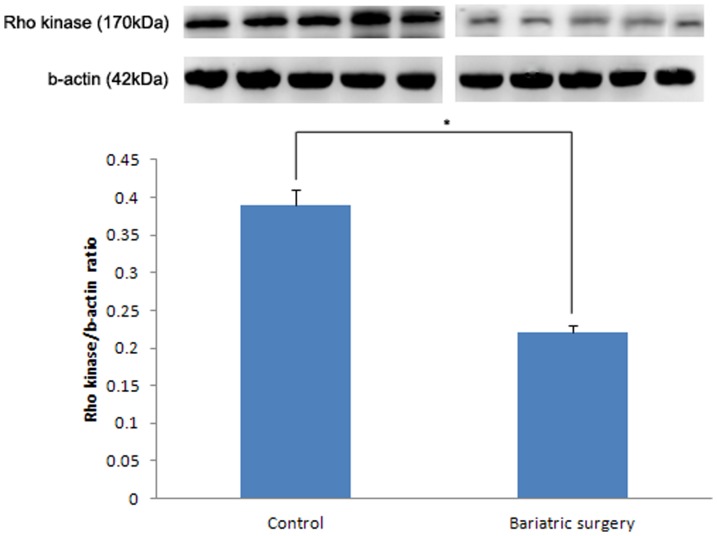
Rho kinase expression in the control and bariatric surgery groups (*: p = 0.032).

Expression levels of eNOS and nNOS was higher in the bariatric surgery group than in the control group. The eNOS/b-actin ratio was found to be 0.28±0.02 and 0.36±0.03 in the control and bariatric surgery groups, respectively (p = 0.027) (Fig. 5). The nNOS/b-actin ratio in the control group and in the bariatric surgery group was 0.32±0.05 and 0.52±0.04, respectively (p = 0.008) (Fig. 6). The Rho kinase expression was reduced in the bariatric surgery group when compared to the control group, and the Rho kinase/b-actin ratio was 0.22±0.03 and 0.39±0.06 in the bariatric surgery group and in the control group respectively (p = 0.032) (Fig. 7).

### Oxidative Stress Analysis

**Figure 8:**
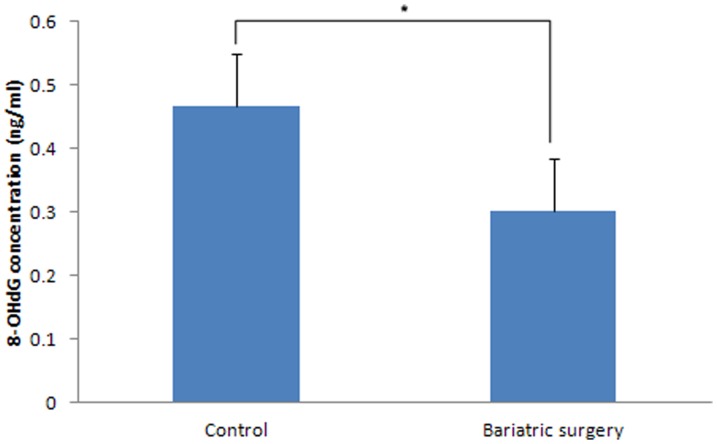
8-OHdG concentration between the control and bariatric surgery groups (*: p = 0.016).

The oxidative stress analysis revealed an 8-OHdG concentration in the corpus cavernosum of 0.301 ng/mL and 0.466 ng/mL in the bariatric surgery and control groups, respectively (p = 0.016) (Fig. 8).

## Discussion

Both forms of diabetes mellitus involve an abnormal carbohydrate metabolism and hyperglycemia. However, the spectrum of diabetic conditions differs in characteristics of insulin production and obesity profile as well as cytokine and lipid levels [9]. Of the two major types of diabetes, T2DM accounts for 90–95% of adult cases of diabetes mellitus in the United States [10].

The clinical manifestation of T2DM is insulin resistance and obesity. Because of these manifestations, T2DM is closely related with systemic diabetic complications such as hypertension, cardiovascular disease, and hyperlipidemia. These complications are sometimes hard to recover from without strict glucose control and proper medication, and may be fatal.

Under sexual stimulation, corpus cavernosum smooth muscle relaxation is initiated and maintained by an increased nitric oxide production through the activation of neuronal nitric oxide synthase and endothelial nitric oxide synthase [11]–[14]. The resulting increased arterial inflow and venous outflow occlusion causes dilation of the cavernosum. Subsequently, the maintenance of intracavernosal pressure creates penile erection. Besides these neuronal and vascular controls of penile erection, the RhoA/Rho kinase pathway also participates in penile smooth muscle tone contraction and relaxation through the intracellular calcium level [15], [16].

In many studies of diabetic rat models, and particularly in the T2DM model, vasodilatory signaling is impaired by a decrease in nNOS activity, non-adrenergic non-cholinergic (NANC) dysfunction, impaired eNOS activation, oxidative stress, cavernosal hypercontractility, veno-occlusive dysfunction, and androgen deficiency. Together, these changes are suggested as the possible mechanisms underlying the development of erectile dysfunction [3], [9].

The obesity pandemic has become a global burden and is now a leading cause of morbidity and mortality. The economic impact of obesity on the United States was approximately $99.2 billion in 1995. The direct economic impact of obesity is similar to that of diabetes [17]. Obese individuals report a high frequency of sexual difficulties [18], [19] often related to a reduced sexual drive and satisfaction, as well as a negative self-image [20], [21]. Non-surgical treatment has been ineffective in producing sustained weight loss among these individuals, and bariatric surgery appears to be the most effective treatment for obesity, particularly among patients with a BMI>40 kg/m^2^ or those with a BMI>35 kg/m^2^ and significant obesity-related comorbidities [22]. Dallal et al. reported that obesity-related sexual dysfunction could be reversible through gastric bypass surgery [19]. However, the histologic and functional evaluations are as yet unknown.

In 1954, an intestinal bypass operation as a potential treatment for obesity was first reported in a study using a dog model [23]. Since then, many researchers have investigated mechanisms by which gastrointestinal bypass causes weight loss, and many have hypothesized malabsorption, caloric restriction, and gastric restriction to be the cause. However, none of these theories have provided sufficient explanation for clinical findings such as how the Roux-en-Y procedure can restore normal blood glucose levels in 84% of patients with obesity-related T2DM [24].

Since this discovery, the relationship between bariatric surgery and glucose homeostasis has generated a tremendous research interest. Because the intestine itself produces, releases, and controls the endocrine signals of energy metabolism, gut hormones such as GLP-1, PYY, and ghrelin were considered responsible for weight loss and the restoration of glucose homeostasis following gastric bypass surgery [22]. As with other theories, however, these theories based on gastrointestinal hormones remain controversial.

In our study, the cavernosal tissue in the control and bariatric surgery groups exhibited differences in biochemical and metabolic activities. In this T2DM rat model study, we have ascertained the effect of duodenojejunal bypass surgery on glucose homeostasis. These metabolic functional restorations are the key factors for micro-, macrostructural, and functional recovery. In the control group, the diminished glucose homeostasis led to structural and microvascular damage. The levels of eNOS and nNOS expression were diminished and Rho kinase expression was increased in the diabetic rats that had received the sham operation, compared to the rats that had received bariatric surgery. These resulted in cavernosal smooth muscle atrophy and vascular dysfunction. With these structural alterations, the functional result should be erectile dysfunction. However, in the bariatric surgery group we observed glucose homeostasis recovery that leads to metabolic and biochemical restoration, an increased level of eNOS, nNOS expression, and a diminished level of Rho kinase expression. This microvascular structural restoration leads to a macrostructural recovery, and thus, results in functional recovery.

Reactive oxygen radicals are responsible for DNA damage, intracellular damage, apoptosis, and subsequently lead to endothelial fibrosis in the corpus cavernosum. 8-OHdG is an established indicator of DNA oxidative stress [25]. In this study, the 8-OHdG level was lower in the rats that underwent bariatric surgery, indicating that bariatric surgery decreases oxidative stress associated with T2DM in the penile corpus cavernosum.

With these metabolic and biochemical alterations, changes in the penile corpus cavernosal structure were observed between the control and the bariatric surgery groups. In the control group, T2DM caused definitive cavernosal fibrosis and a reduced amount of smooth muscle. In comparison, the rats underwent bariatric surgery had relatively higher proportions of smooth muscle fibers in the cavernosal tissue. This appears to indicate that bariatric surgery could potentially reduce cavernosal fibrosis in the setting of diabetes. Through these findings, bariatric surgery seems to improve glucose homeostasis with biochemical factors, thus leading to structural and functional improvements in the corpus cavernosum and resulting in improved erectile dysfunction.

The limitations of this pilot study included firstly the relatively small sample size and lack of metabolic parameters. As the purpose of this experiment was to identify the structural and functional background of bariatric surgery in cases of diabetes, we did not perform metabolic evaluations such as testosterone and lipid profiles. In future studies, evaluation of these metabolic parameters can be further investigated. Second, the lack of physical measurement, except that of body weight, means that the results of this study cannot be generalized to human studies because obesity is closely correlated to BMI and abdominal circumference. Further evaluation with a larger sample size and metabolic parameter evaluation would be needed. A conventional drug treatment model with a positive control would be needed to clarify the effect of bariatric surgery.

Third, the baseline information of histological/expression study were not evaluated. With baseline information for evaluating the pre- and post-operative changes in histological and expression study, the exact effect of bariatric surgery on erectile tissue might be more elucidated. Though this experiment focused on the comparison of the effect of bariatric surgery on erectile dysfunction in a T2DM rat model, the lack of baseline information of histological and biochemical is limitation of this study. In a future study, the limitations including baseline data should be evaluated to understand the exact effect of bariatric surgery on erectile tissue.

## Conclusions

Bariatric surgery restores glucose homeostasis. Restoration of glucose homeostasis induces eNOS and nNOS expression and reduces Rho kinase expression in the corpus cavernosum of the T2DM rat. This metabolic recovery induces cavernosal microvascular and structural changes thus resulting in erectile functional recovery.
